# Tics: neurological disorders determined by a deficit in sensorimotor gating processes

**DOI:** 10.1007/s10072-022-06235-0

**Published:** 2022-07-04

**Authors:** Edoardo Dalmato Schilke, Lucio Tremolizzo, Ildebrando Appollonio, Carlo Ferrarese

**Affiliations:** 1 Neurology Unit, ASST Monza Ospedale “San Gerardo”, Via Pergolesi 33, 20900 Monza, MB Italy; 2 School of Medicine and Surgery, University of Milano-Bicocca, Milan, Italy

**Keywords:** Tic, Prepulse inhibition of startle, Sensorimotor gating, CSTC loops

## Abstract

Tic related disorders affect 4–20% of the population, mostly idiopathic, can be grouped in a wide spectrum of severity, where the most severe end is Tourette Syndrome (TS). Tics are arrhythmic hyperkinesias to whom execution the subject is forced by a “premonitory urge” that can be classified as sensory tic, just-right experience or urge without obsession. If an intact volitional inhibition allows patients to temporarily suppress tics, a lack or deficit in automatic inhibition is involved in the genesis of the disorder. Studies have assessed the presence of intrinsic microscopic and macroscopic anomalies in striatal circuits and relative cortical areas in association with a hyperdopaminergic state in the basal forebrain. Prepulse inhibition (PPI) of the startle reflex is a measure of inhibitory functions by which a weak sensory stimulus inhibits the elicitation of a startle response determined by a sudden intense stimulus. It is considered an operation measure of sensorimotor gating, a neural process by which unnecessary stimuli are eliminated from awareness. Evidence points out that the limbic domain of the CSTC loops, dopamine and GABA receptors within the striatum play an important role in PPI modulation. It is conceivable that a sensorimotor gating deficit may be involved in the genesis of premonitory urge and symptoms. Therefore, correcting the sensorimotor gating deficit may be considered a target for tic-related disorders therapies; in such case PPI (as well as other indirect estimators of sensorimotor gating) could represent therapeutic impact predictors.

## TIC-related disorders and Tourette Syndrome

Tic disorders affect 4–20% of the population [1]. Most cases are classified as idiopathic; they can be grouped in a wide spectrum of severity, where the most severe end is represented by Tourette Syndrome (TS) that has a lifetime prevalence of 0.5–0.7% [2]. Tics are arrhythmic hyperkinesias, described as sudden, rapid movements or vocalizations, characterized by a different grade of complexity (in terms of number of muscular groups involved), and enhanced by emotive factors and fatigue [3]. They are typically stereotyped, fluctuating, distractible and suggestible [4].

The onset of TS is typically in childhood (the majority between the ages of 4–6 years). Tic severity typically peaks in adolescence (the majority between the ages of 10–12 years). In most cases, symptoms are fluctuating, following a waxing and waning course. Even if they have a fluctuating course, from the onset to the peak of the disease the symptoms tend to aggravate. The most common tics at onset are simple motor tics [5]. The appearance of additional tics typically progresses in a rostral-caudal pattern, involving trunk and limbs, with a simultaneous increase of the number of muscular groups involved. Vocal tics tend to manifest only in advanced states of the disease [6].

Most cases will show an improvement of symptoms by late adolescence or early adulthood [7]. If in less than 1/3 of individuals there will be a complete remission of symptoms, the remainder will have mild, often barely noticeable symptoms, except for a 10–20% of cases that will continue to have significant symptoms [8].

All idiopathic tic-related disorders show an association with psychiatric disorders. This is particularly true for TS. Only approximately 20% of TS cases occur in absence of a significant comorbid diagnosis [9]. The most represented comorbidities are obsessive–compulsive disorder (OCD), and attention deficit-hyperactivity disorder (ADHD), which occur in 60% of individuals [10]. Less common, but still more frequent than in the general population are autism spectrum disorders, depression, anxiety and behavioural disorders [10].

Tics are generally considered movements to whom execution the subject is forced by a “premonitory urge” [6]: sensation of discomfort that tic emission helps to alleviate or discharge. Premonitory urges can be classified as sensory tic, just-right experiences or urges (without obsessions) [11].

Sensory tic is a bodily sensation, mainly musculoskeletal, perceived as uncomfortable. A just-right experience is an inner discomfort produced by perception that elements of the surrounding environment (sounds, objects, people, movements, actions) are not harmonized in a certain “right way.” This experience has been compared to a compulsion [12], even if is more closely related, by a biological point of view, to tic-related disorders than to OCDs [13]. An urge without obsession is a drive to do a certain action associated with neither sensory phenomena nor a sense of disharmony of the surrounding environment.

## CSTC loops abnormalities in tic-related disorders

**Fig. 1:**
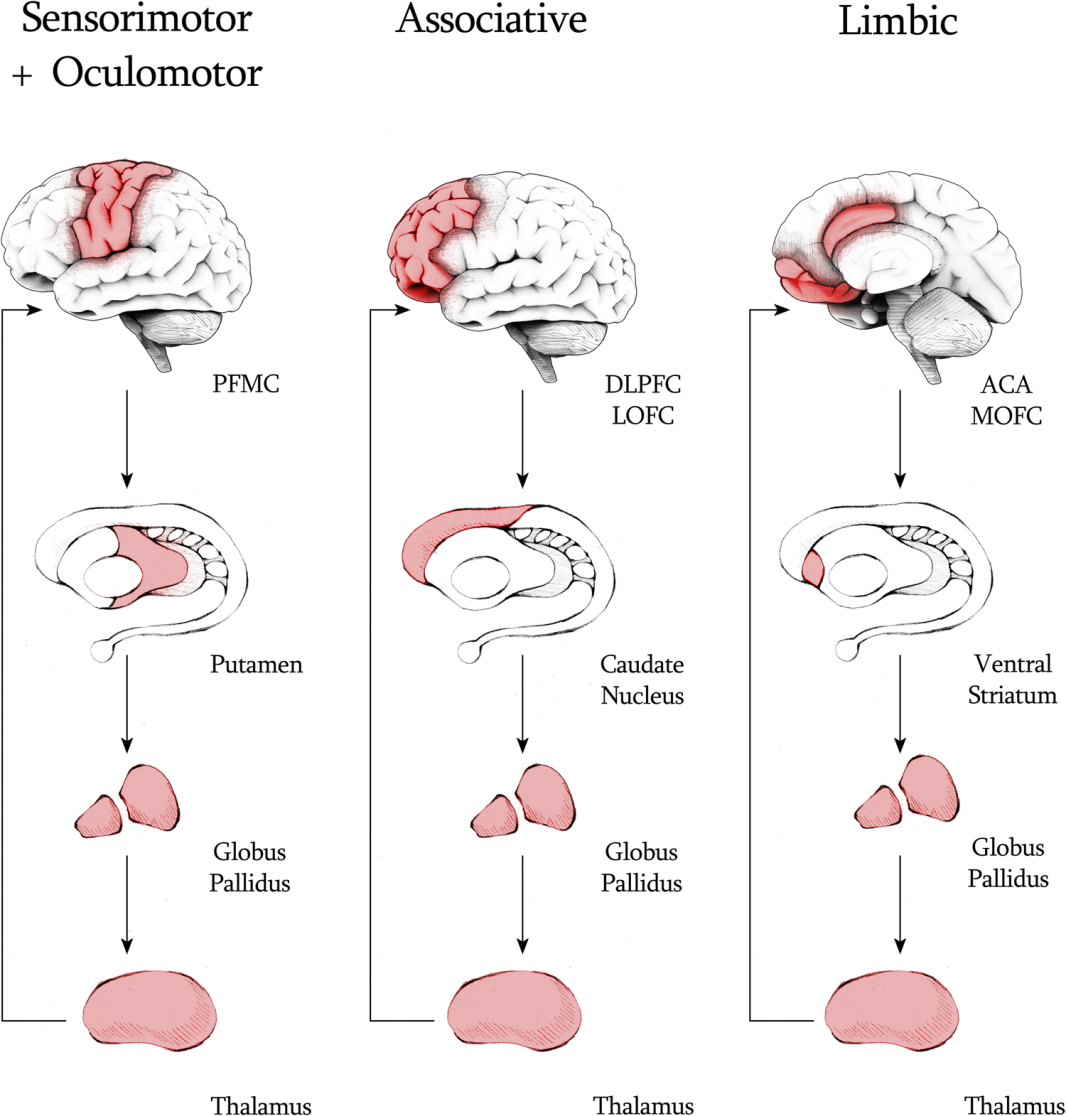
A common partition of the cortico-striato-thalamo-cortical (CSTC) loops is into three major domains: sensorimotor + oculomotor, associative and limbic. Each domain relates with approximate cortical and striatal areas. The sensorimotor domain is made by loops mainly involving the prefrontal motor cortex (PMFC), and the putamen. The associative domain is made by loops mainly involving the dorsolateral prefrontal cortex (DLPPC), the lateral orbitofrontal cortex (LOFC), and the dorsal portion of the caudate nucleus. Finally, the limbic domain is formed by loops mainly involving the medial orbitofrontal cortex (MOFC), the anterior cingulate cortex (ACA), and the ventral portion of the striatum, but also subcortical elements of the limbic system, such as accumbens nucleus and basolateral amygdala (not shown). Tic-related disorders have been associated with a dysregulation primarily of the sensorimotor and oculomotor loops

The limbic, associative and sensorimotor domain of cortico-striatal-thalamo-cortical (CSTC) loops are composed of multiple largely parallel (but partially overlapping) circuits that direct information from cerebral cortex to basal ganglia and thalamus, and then back again to specific region of the cortex (Fig. 1). Dopamine is a crucial regulator of striatal microcircuitries. Dopaminergic neurons in the ventral tegmental area (VTA) preferentially innervate the nucleus accumbens, while dopaminergic neurons in the substantia nigra mainly project to the dorsal striatum [14].

Double-blind clinical trials in which neuroleptics, that preferentially block dopaminergic D2 receptors (such as haloperidol and pimozide), have been found to be effective in the temporary suppression of tics [15], as well as data from animal models, in which the administration of tetrabenazine (inhibitor of the uptake of DA into synaptic vesicles) resulted effective in tic suppression [16], have posed the basis for a “dopamine hypothesis”: tic disorders can be related with an excess of nigrostriatal dopaminergic activity. This hypothesis is supported by in vivo neuroimaging studies that documented increase of dopamine transporter (DAT) binding in the neostriatum and increase of DA storage and DA release in the ventral striatum [17].

Most neurons (> 90%) in the striatum are γ-aminobutyric acid-ergic (GABAergic) medium spiny neurons (MSNs). They are activated by motor behaviours elicited by memory-encoded or environmental cues, exhibiting a context-dependant firing pattern [18]. Whitin the striatum MSNs form a weak lateral inhibitory network (feedback inhibition), important in regulating striatal output, while GABAergic interneurons, despite in much smaller numbers, exert a powerful control over striatal excitability (feedforward inhibition), modulating MSNs excitability. Feedforward inhibition is mediated by fast-spiking GABAergic interneurons (FSINs) [19]. FSINs receive direct cortical inputs predominantly from lateral cortical regions, including the primary motor and somatosensory cortex. These interneurons interact with adjacent MSNs. It does appear that FSINs are activated in a coordinated fashion just prior to a decision being made in a striatal-dependent task [20]; once activated, they determine a synchronous inhibition of many adjacent MSNs via synapses on cell bodies and proximal dendrites. A second class of GABAergic interneurons comprises the neuropeptide Y (NPY), nitric oxide synthetase and somatostatin expressing interneurons. These cells present a least dense axonal arborization and electrophysiologically characterized by low threshold calcium spikes [21].

Dopaminergic afferences exert an influence also on GABAergic interneurons activity via presynaptic and postsynaptic actions. Dopamine release induced by drugs (such as methamphetamine) increases the activity, while dopamine D2 receptor antagonist depresses the firing frequency of FSINs. FSINs activity is enhanced directly by postsynaptic D5 receptors and indirectly by reducing GABAergic inputs to FSINs via presynaptic D2 receptors [22, 23]. Inhibition of the nigrostriatal circuit causes impaired and poorly timed synchronization of FSINs activity [24]. On the other hand, repeated administration of methamphetamine enhances preproNPY mRNA expression in a D1-dependent manner. Low threshold interneurons are depolarized by dopamine throughout D1 receptors [25]. While dopamine depletion promotes an oscillatory activity in FSINs, a dopaminergic excess promotes a tonic activity, both conditions may impair MSN activity and consequentially corticostriatal encoding [26]. Furthermore, it has been showed that endogenous dopamine influences striatal microcircuitries by negative regulating the number of tyrosine-hydroxylase (TH) expressing MSNs, whom TH immunoreactive axons interact with proximal dendrites and soma of NPY expressing interneurons [27].

FSINs have been widely implicated in studies regarding reward-seeking since activation of FSINs have been showed to result in behavioural changes to reward. One study, manipulating neuronal firing of FSINs and assessing throughout microdialysis the changes in GABA and glutamate levels, showed that FSINs activity influences the initial expression of reward conditioned responses, and that their influence on MSNs declines with training. Moreover, FSINs activity can mediate learning by enhancing performance during associative learning [28].

The striatum also presents cholinergic tonically active neurons (TANs). Both TANs and dopaminergic neurons emit robust signals following reward-related events, signals involved in the calculation of perceived salience of numerous perceptual cues arriving to the striatum. It has been postulated that dopamine (DA) and acetylcholine (ACh) act antagonistically in the striatum, having opposing effects on the excitability of MSNs [29]. Both striatal DA and ACh affect plasticity of cortico-striatal transmission, therefore they are both crucial in learning processes [30].

In monkeys performing probabilistic instrumental conditioning tasks, although different events yielded responses with different latencies, the response of the two populations coincided, indicating integration at the target level, yet while the DA neurons’ response reflects mismatch between expectation and outcome, TANs activity is invariant to reward predictability. Therefore, is likely that striatal cholinergic and dopaminergic systems carry distinct messages by different means, which can be integrated differently to shape the basal ganglia response to reward-related events [31].

Not only dopaminergic and cholinergic afferences act antagonistically on MSNs, but also dopamine modulates the activity of TANs: stimulation of dopaminergic axons in dorsal striatum modifies ionic conductance in TANs, suggesting that dopamine dynamically controls cholinergic tone. Reciprocally, TANs interneurons modulate dopamine output thought activation of both muscarinic and nicotinic cholinergic receptors on dopamine terminals [32].

Neuropathology studies of post-mortem TS brains documented a greater than 50% reduction in the FSINs population in the caudate nucleus and a 30–40% reduction of these same cells in the putamen [33]. A more recent post-mortem study confirmed a 50–60% decrease of both FSINs as well as a loss of TANs in the caudate nucleus and putamen; in addition, TANs were decreased in TS patients in the associative and sensorimotor regions, but not in the limbic regions of the striatum, such that the normal gradient in density of cholinergic cells (highest in associative regions, intermediate in sensorimotor and lowest in limbic regions) was abolished [34].

The complex interaction of motor and psychiatric symptoms and evidence from neuroimaging indicate that tic-related disorder pathophysiology involves CSTC networks that span different functions, including sensorimotor, limbic and associative networks.

Volumetric magnetic resonance imaging (MRI) studies showed an approximate 5% reduction in caudate volume in TS patients [33], data that fits the microscopical loss of interneurons. It was also found an inverse correlation between caudate volume in childhood and tic severity in adulthood [35]. Cortical thickness has also been measured comparing TS-affected children/adolescents with age- and sex-matched healthy controls, showing a cortical thinning most evident in the regions of the sensory and motor cortex. Thinning in these regions directly correlated with tic severity [36]. Cortical thinning was also found in other regions, including lateral orbitofrontal and dorsolateral cortex. This data could correlate with an increased vulnerability to psychiatric comorbidities, such as OCD.

In one study, using functional magnetic resonance (fMRI), the spatiotemporal pattern of coactivation of areas within the motor cortex during tics was contrasted with that seen in healthy control subjects during matched, intentional movements. The supplementary motor area (SMA) showed a significant broader profile of cross-correlation to the motor cortex during tic rather than intentional movements, highlighting a potential importance of the SMA hyperactivity in tic generation [37]. In another study, tic behaviour was highly correlated with an increased activity in a set of areas, including not only SMA but also premotor, anterior cingulate, dorsolateral-rostral prefrontal, primary motor cortices, Broca’s area, insula, claustrum. Additionally, a fluorodeoxyglucose (FDG)-PET study reported differences within the connectivity within these areas and the basal ganglia (especially the ventral striatum) [38]. By distinguishing a frontoparietal network (likely involved in rapid and adaptative control of movements) and a cingulo-opercular network (apparently significant for set maintenance), it was found that adolescents with TS had immature patterns of connectivity, particularly within the frontoparietal network; additionally, aberrant connections were also documented in brain regions of the frontoparietal network [39].

Deep brain stimulation (DBS) of centromedial thalamus and globus pallidus pars interna (GPi) have been tested in some randomized controlled trials (RCTs) of small cohorts of TS patients. Some of these RCTs reported a significant and long-term reduction of tic during active DBS compared to shame [40–42]. However, DBS outcomes vary substantially across patients and reliable predictors of therapeutic responses have not been identified yet [43]. Studies reported that a structural connectivity of the site of stimulation in centromedial thalamus and GPi to the specific components of the frontostriatal, limbic and motor networks were correlated with tic improvement, thus suggesting that the connectivity profile of the stimulation site may be related to clinical outcomes of DBS in tic-related disorders [44–46]. According to these previous studies, Johnson et al. [47], using tractography maps, showed that DBS of the GPi may be associated with tic improvement following modulation of limbic, associative networks and the efficiency was also correlated with a higher connectivity to caudate, thalamus and cerebellum, while modulation of sensorimotor and parieto-temporal-occipital networks and a higher connectivity to putamen and cerebellum may be responsible of tic improvement following DBS of the centromedial thalamus. According to the authors, the stimulation of GPi may improve tics by decreasing activity in downstream limbic and associative tic-related networks, while stimulation of centromedial thalamus may improve tics by directly disrupting local tic-related pathological activity.

McCairn et al. [48], after obtaining a pharmacologic motor tic animal model by microinjecting a GABA-antagonist within the sensorimotor striatum: putamen, proposed the hypothesis that tics are generated by a dynamical process that involves an abnormal reciprocal interaction between cortex and basal ganglia, in which an abnormal dopamine burst represents a necessary condition for motor tic production. It is conceivable that an abnormal dopamine efflux alters the typical selection processes implemented by the CSTCs loops of selecting intrinsic noise and inputs received by the system from cortical regions, making it overly sensitive to the received signals so that spurious primary cortex activations result to be disinhibited.

In patients treated with DBS, both GPi and centromedial thalamus stimulation capacity to reduce tics were correlated with a higher connectivity of the stimulated areas to cerebellum, suggesting the presence of an aberrant cerebellar cortex activity during tic production through dysinaptic link connecting the basal ganglia to the cerebellum [49]. These links are dysinaptic since it has been described the interposition of pontine nuclei between the subthalamic nucleus and the cerebellar nuclei, and excitatory since most of the pontine neurons projecting to the cerebellum are glutamatergic. Once activated, the cerebellum might feedback the basal ganglia and directly affect the descending motor pathways [50]. The augmented activity of the cerebellum may determine a further increase of the sensitivity of the selection process within the basal ganglia-thalamo-cortical system and spurious activations of primary motor cortex may be selected more easily and develop into motor tics. Further studies are needed to prove this hypothesis and to prove an effective role of an abnormal cerebellar activity in tic production.

In the genesis of the abovedescribed anomalies within the CSTCs loops may be involved neuroinflammatory processes. Lennington et al. [51] compared basal ganglia transcriptome by RNA sequencing in the caudate and putamen of 9 TS with 9 matched normal confronts, finding 309 down-regulated and 822 upregulated genes in TS individuals. The 9 TS patients did not meet criteria for Pediatric Autoimmune Neuropsychiatric Disorder Associated with Streptococcal infection (PANDAS) or Pediatric Acute onset Neuropsychiatric Syndrome (PANS). However, in addition to expected evidence of metabolic alterations of striatal interneurons (FSINs and TANs) that may be linked to their death and dysfunction, they found a significant increase in immune and inflammatory transcripts that appear to be endogenous to the CNS. It remains unclear whether the interneurons death is secondary to neuroinflammation or neuroinflammation is caused by the interneuron’s death. An association of symptoms onset or exacerbation of tic disorders with acute infections has been documented [52]. Further studies are necessary to redeem the exact role of neuroinflammation in tic-related disorders.

## Prepulse inhibition

Prepulse inhibition (PPI) of the startle reflex is a measure of inhibitory functions by which a weak sensory stimulus (prepulse) inhibits the elicitation of a startle response caused by a sudden intense stimulus (pulse). It is viewed as an operational measure of a neural process called “sensorimotor gating” by which unnecessary stimuli are eliminated (or “gated out”) from awareness, so that an individual can focus attention on the most salient aspects of the surrounding environment [53].

**Fig. 2:**
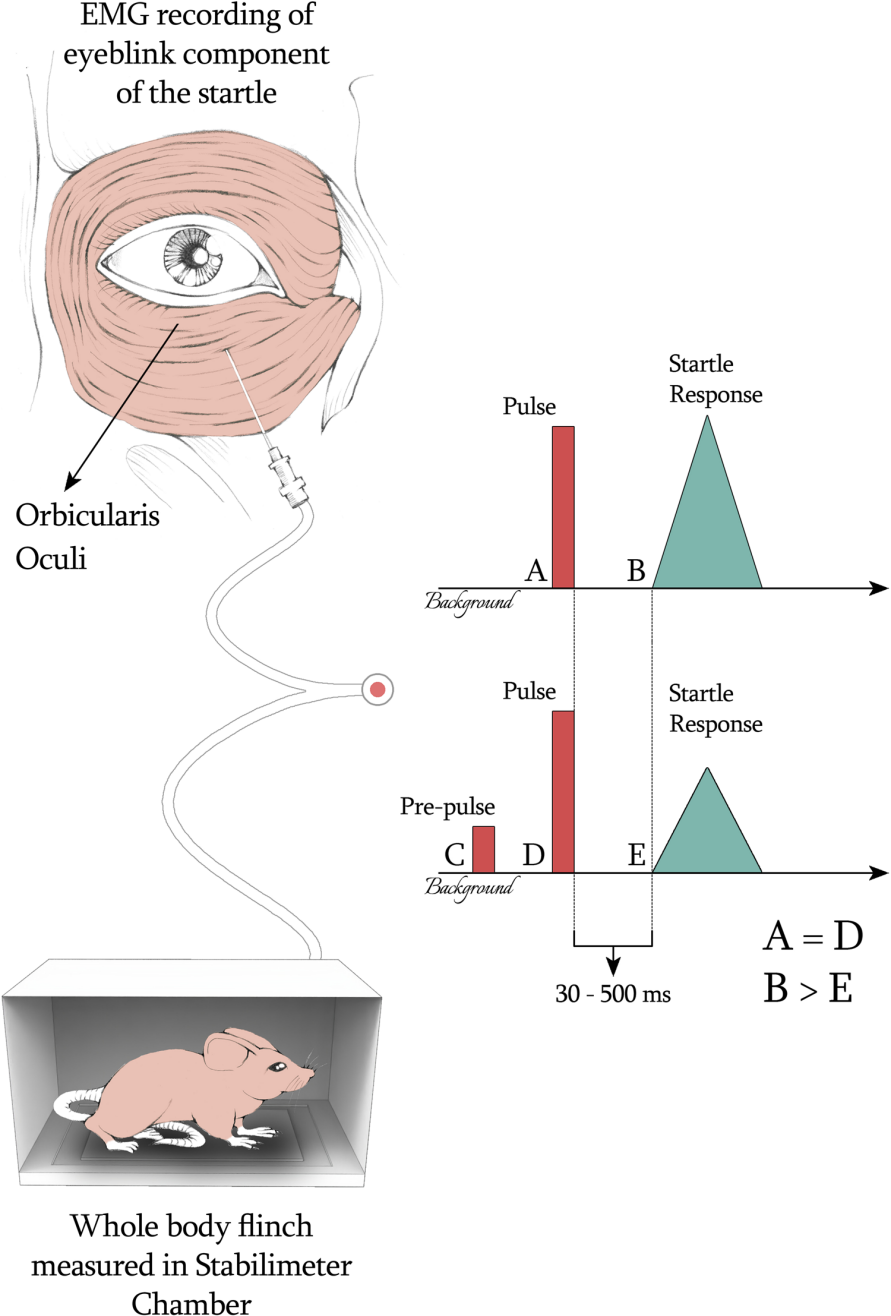
Prepulse inhibition (PPI) is considered an operational measure of “sensorimotor gating” a neural process aimed to eliminate unnecessary stimuli from awareness. PPI can be measured through the inhibitory effect exercised on the startle reflex by a prepulse (administrated 30-500 ms before an intense stimulus). The intense stimulus and relative pre-pulse may be presented across multiple modalities (visual, auditory or tactile). In humans, it is possible to evaluate the startle reflex by studying the eyeblink component of the startle throughout electromyography (EMG). The startle reflex can also be evaluated in rats and mice using stabilimeter chambers capable to measure the whole-body flinch elicited by intense stimulation

The startle reflex consists of a contraction of skeletal and facial muscles in response to a sudden intense stimulus that may be presented by across multiple modalities (visual, auditory or tactile). PPI is the normal suppression of the startle reflex that occurs when the intense startling stimulus is preceded by a relatively weak stimulus. In order to inhibit startle, the prepulse must precede the pulse by 30–500 ms [54]. The major advantage of the PPI of the startle reflex is that it can be studied across species, leading to translational research opportunities [53, 55, 56]. In humans, it is possible to evaluate the eyeblink component of the startle by using electromyography (EMG) of the orbicularis oculi muscle; in rats and mice, Stabilimeter Chamber measures the whole-body flinch elicited by stimuli that are similar (or identical) to those used in humans [54] (Fig. 2).

Different theories about how information is processed have been formulated. One first theory affirmed that incident stimuli are processed in a sequential manner through a series of “stages” that occur at progressively higher sites in the central nervous system. Abnormalities of this process were conceptualized as potentially occurring at various stages resulting in a heterogenous spectrum of cognitive deficiencies and associated behaviours. For example, according to this theory, psychotic symptoms were viewed as a result of a deficit in early stages of information processing. Such deficit was thought to cause a cascade of unnecessary information at downstream stages that ultimately resulted in an abnormal cognitive integration [53].

The “stages” model has been increasingly challenged by an “integrationist” model that relies on neural networks theory: the processing of incident stimuli is operated not only by a filter action within the progressively higher sensitive sites but also by the integration of a time-coordinated activity of neurons in multiple sites at different levels of the nervous system [57].

This integrated, multisite activity is thought to be fundamental for the “sensorimotor gating,” a neural process intended to help the organism regulating environmental inputs in order to allocate attentional resources to salient stimuli [58]. The specific characteristic of an individual’s gating process is thought as being plastic and influenced by a combination of genetic traits [59] and environmental factors such as neonatal insult [60] and social isolation [61] as well as toxic influences of neurochemical and hormonal milieu of the nervous system [62].

Studies have proved a PPI disruption in several psychiatric disorders. PPI has been particularly valuable for studying the neurobiology of schizophrenia and related spectrum disorders since gating deficit is an important feature of pathophysiology of the disease and deficits in gating of cognitive and sensory information are particularly relevant clinical features of the disorder [59].

Different data suggest that CTSC loops are involved in the modulation of PPI: a loss of healthy neural communication between limbic and basal ganglia structures results in PPI deficits, underlining the existence of cortico-striato-pallido-pontine (CSPP) circuits [63]. Manipulations of the hippocampus and basolateral amygdala, as well as dysfunctions in the medial prefrontal cortex and the orbitofrontal cortex, can lead to changes in PPI. While it was originally thought that the hippocampus acts on PPI modulation via the nucleus accumbens, recent anatomical and functional data suggest that the efferent connections responsible for PPI disruption are directed from hippocampus to medial prefrontal cortex [64].

There is also evidence pointing out that dopamine receptors within the striatum nucleus plays out an important role in PPI modulation: in preclinical studies, the activation of dopamine receptors by either direct agonist (such as apomorphine) and indirect agonist (such as amphetamine) leads to robust deficit of PPI [55]. In human, volunteers were tested PPI after administration of the D2 receptor antagonist (and probable D1 receptor partial agonist) bromocriptine (1.25 mg), the receptor antagonist haloperidol (3 mg), and a combined treatment with the cited doses of the two drugs in a balanced double-blind protocol. In all cases, the tested prepulse-to-pulse interval was 120 ms. The baseline startle response was not altered by any of the pharmacological treatments and PPI was observed to be appropriately dependent upon the prepulse intensity. In all conditions, bromocriptine robustly reduced PPI. However, somewhat paradoxically, the dopamine antagonist haloperidol also elicited a much smaller, albeit statistically significant, decrement in PPI [65]. A second study by the same group confirmed similar findings about bromocriptine effect on PPI but failed to replicate the effect of haloperidol [66]. Therefore, the haloperidol effect on PPI seems not to be reliable in humans. However, the most significant data that emerged in these two studies was that haloperidol is effective in antagonizing the PPI-disruptive effect of bromocriptine. The same result was also found in animals in another study [55]. Moreover, evidence suggests that GABAergic system participate in determining PPI. Using magnetoencephalography, Inui et al. [67] evaluated the inhibition on acoustic startle determined by weak stimuli presented at 10–800 ms before the startle, showing the existence of two distinct inhibition peaking at approximately 20–60 ms and 600 ms that appear to reflect inhibitory post-synaptic potentials (IPSPs) of FSINs and cortical somatostatin-positive interneurons (Martinotti cells). In another study [68], the inhibition occurring at 20–60 ms has been furtherly divided in two components, since the administration of diazepam enhances the inhibition at 10–20 ms, while the administration of baclofen enhances the inhibition at 40–50 ms. The fact that the short-latency component is elicited by diazepam suggests the involvement of GABA_A_ receptors, while the effect of the baclofen on the further component suggests the involvement of GABA_B_ receptors.

## Prepulse inhibition in tic-related disorders

Tics can be transiently suppressed by volitional effort or will. This concept has led to the hypothesis that tics result from a failure of motor systems’ tonic inhibition mechanisms. It has also been hypothesized a deficit in mechanism of automatic inhibition [69]. The brain is subjected to a continuous flow from the external environment of potential triggers to specifical movements. These potential movements are automatically suppressed by a process non subjected to voluntary control [70].

In one study [71], volitional inhibition was evaluated by conditional stop signal task (CSST) and automatic inhibition by using the masked priming task (how the reaction time to imperative stimulus is affected by the presence of an unperceived priming cue), excluding deficits in the volitional inhibition and outlining an altered automatic inhibition. This study suggests that intact volitional inhibition allows patients to voluntarily suppress their tics, while a lack or deficit in automatic inhibition is involved in the genesis of the tic disorder. PPI is an operational measure of sensorimotor gating, a process of central inhibition of unnecessary incident stimuli. It can be considered an automatic process, apart from a volitional component correlated with sensitive attention. At the same time, a lack or deficit in automatic inhibition is involved in the genesis of tic-related disorders. Thus, it is conceivable that the pathological process responsible for the loss of sensorimotor gating in tic disorders may determine the genesis of the premonitory urge and symptoms.

Studies showed that TS patients exhibit a blunted PPI. In a first study, utilizing supraorbital electrical nerve stimulation to produce adequate blink responses and measuring the decrease in amplitude resulting from electric prestimuli just above sensory threshold, 7 boys comorbid for ADHD and tic disorder had significantly reduced PPI compared to 14 screened controls and seven boys with ADHD alone [72]. A second study applied a “fMRI friendly” protocol to detect PPI in TS children: bilateral eyeblink PPI was tested using chin air puffs to elicit startle and prepuffs to the dorsal hand surface as inhibiting stimuli; compared to control subjects, TS children exhibited comparable startle magnitude and habituation, but significantly reduced PPI after prepuffs [56]. Following a similar protocol to evaluate PPI inhibition, in another study, whole-brain fMRI was taken in 17 TS adults and 16 healthy controls in order to evaluate the neural correlates of PPI in adult TS subjects. A normal level of PPI in healthy controls and a considerably lower in TS patients were observed, even if the difference did not reach statistical significance. In healthy subjects, PPI was associated with increased activity in multiple brain regions and group comparison identified 9 regions where brain activity during PPI differed significantly between TS and healthy controls; among these 9 regions, regression analysis demonstrated a significant positive linear relationship between the current tic severity (measured by Yale Global Tic Severity Scale) and the activation of left caudate (while the right caudate only approached statistical significance) [73]. This study supports the abovementioned role of an abnormal caudate nucleus functioning in the development of tic disorders and suggests that such functioning could alter sensorimotor gating mechanisms.

PPI is not the only means of investigating sensorimotor gating. Another mean is assessing sensorimotor gating by event-related potentials (ERPs) paradigms utilizing paired stimuli to test habituation, strategy that grants a remarkable temporal precision. Such type of studies has consistently identified a diminished amplitude of the contingent negative variation (CNV) component, which can be considered as a marker of cognitive anticipation for a forthcoming sensory event, in both adults and children affected by TS [74]. Additionally, patients with more severe tics have lower CNV amplitudes, indicating a reduced pre-conscious capacity to anticipate environmental stimuli [75].

There is also a report affirming that TS patients endorse difficulties in sensory gating processes, specifically the ability to modulate stimulus intensity preventing perceptual inundation and the ability to focus attention or prevent distractibility. It was also assessed a low threshold of perception causing over-inclusion and distractibility, a vulnerability to perceptual and attentional anomalies during periods of fatigue and stress [76]. Another work reported complementary findings but also reported a heightened sensitivity to sensory stimulation in 80% of the tested TS patients that was not accompanied by significant differences of tactile and olfactory thresholds compared to control subjects. The authors concluded that subjective sensitivity differences in TS patients reflected altered central information processing rather than enhanced peripheral sensory detection [77].

Turning to internal stimuli, it remains uncertain whether TS sensory gating dysfunction affects interoceptive stimuli. In one study among 12 adult TS subjects (without psychiatric comorbidities) and 12 healthy controls, magnetoencephalography was used to study volitional finger movement in two task conditions: a self-paced and an externally triggered task. If motor field amplitudes did not differ between the two groups, in both tasks, motor field peak amplitudes were increased in TS patients. Moreover, larger motor evoked field amplitudes during self-paced movements were inversely correlated with motor tic frequency and severity, suggesting the presence of an overflow of interoceptive stimuli determined by an altered subcortical gating [78]. Further experiments are needed to confirm this interpretation.

## Tic therapies and prepulse inhibition

As mentioned, effective sites for DBS in TS patients are GPi and centromedial thalamus. One study demonstrated that high frequency DBS of the thalamic centromedian parafascicular nucleus and of the entopeduncular nucleus (EPN) in mice (EPN can be considered a rodent analog of the human GPi) prevented the PPI disruptive effects of apomorphine, a dopaminergic agonist [79]. However, the effect of DBS on PPI in humans has not been proved yet. In a recent study, PPI acoustic startle reflex was measured in TS patients, treated with DBS of the centromedian and ventro-oral internal thalamic nucleus and the anterior limb of internal capsule-nucleus accumbens area respectively, and aged-/gender-matched healthy controls. PPI of the DBS groups was measured in randomized order in ON and OFF stimulation condition. No significant differences were found in PPI (%) of patients with TS between ON and OFF condition. However, if PPI levels were significantly reduced in TS patients in the ON condition compared to heathy controls, no significant difference was found in PPI between TS in the OFF condition compared to healthy controls [80].

Among all of TS therapeutics to date, Habit Reversal Therapy, also termed as Comprehensive Behavioural Interventions for Tics (CBIT), has been proved to have a comparable, if not better, efficacy to other known tic medications in both TS patients [81]. CBIT relates heavily on the ability to initiate an appropriate preventative response after detecting the premonitory urge. This process is very similar to that used in cognitive and behavioural therapy (CBT) where individuals identify an event (such as a delusion, obsession) and initiate a cognitive and behavioural process in order to interrupt the consequences of the event (such as avoidance, compulsion). The purpose is to render over time the volitional components of this inhibitory process more automatic, in other words to shift the regulation of this process from cortical to subcortical circuitries. CBT is used in schizophrenia, where PPI disruption has been assessed as a predictor of the therapeutic impact of CBT [82]. Therefore, it is possible to assert that PPI could represent a therapeutic impact predictor also in CBIT in tic-related disorders.

Finally, the sensorimotor gating intrinsic to tic-related disorders suggests the possibility of an enhancement of the CBIT approach by administration of “pro-cognitive therapies” [83] for augmenting sensorimotor gating processes that would potentiate the ability to assert volitional control over semiautomatic motor response. It has been proved that, especially in individuals with low levels of PPI, different drugs can enhance PPI, such as atypical antipsychotics, the cathechol-O-methyl-transferase inhibitors, tolcapone, the low potency NMDA antagonist, memantine.

After a study showed that 5alpha-reductase inhibitor finasteride could reduce tic severity in 10 adult TS male patients [84], PPI was utilized to explicate such result: in rats finasteride could reduce the PPI disruptive effect of dopaminergic agonists after systemic, intraventricular and intracerebral administration. However, the administration of the same drug in other brain regions failed to replicate the same result, thus suggesting that the finasteride effect on PPI reflects the action on the cells of the ventral striatum [85]. This study suggested that PPI may be considered as an effective marker to test the effectiveness of treatment in tic-related disorders.

Using a D1CT-7 mice, a well-characterised animal model of TS, it has been proven that acute environmental stress is capable to exacerbate tic-like responses and cause sensorimotor gating deficits (measured by PPI). These phenomena were associated with an increase in plasma corticosterone as well as cortical neurosteroids, such as allopregnanolone. The effects of allopregnanolone on the GABA-A receptors are antagonized by its 3beta-epimer, isoallopregnanolone [86]. In a recent study the administration of isoallopregnanolone in D1CT-7 mice not only produced a significant reduction in tic-like behaviours but also significantly reversed the PPI disruptive effect produced by stress [87]. Given recent evidence that positive allosteric modulators of GABA-A receptors containing alpha6 subunities oppose the behavioural effects of dopamine, in D1CT-7 mice was also tested of DK-I-56–1, a highly selective positive allosteric modulator of GABA-A receptors containing alpha6-subunities. DK-I-56–1 significantly reduced tic-like behaviours and PPI deficit in the transgenic mice; DK-I-56–1 also prevented the exacerbation of spontaneous eyeblink reflex induced by potent dopamine D1 receptor antagonist SKF 82,958, a proxy for tic-like responses [88].

## Conclusions

The precise pathophysiologic mechanism of tic-related disorders remains unclear, but a CSTCs circuit dysfunction is likely to be involved. It has been documented a significant reduction in interneuronal population in the striatum associated with an aberrant neurotransmitter functioning, including dopamine and GABA. Such features on a microscopic scale coexist on a macroscopic scale with network abnormalities within CSTCs loops documented by volumetric and functional MRI studies, PET studies and studies that associated DBS with tractography maps. The dysfunction of striatal interneurons and the network abnormalities observed within the CSTCs loops may be considered candidate mechanistic link in tic-related disorder pathophysiology.

Tic-related disorders may be also characterized by a sensorimotor gating deficit, which may be determined by the described anomalies within the CSTC loops. Sensorimotor gating is the suppression of irrelevant information to ensure the ability to focus on relevant stimuli; it can be measured using prepulse inhibition (PPI) of the startle reflex. If sensorimotor gating is truly impaired in tic-related disorders, PPI and others indirect indicators of sensorimotor gating may become a marker to test the effectiveness of actual and future treatments.

Notably, a GABAergic interneurons dysfunction is postulated to underlie several neurodevelopmental disorders, including OCD, autism and schizophrenia, all of which exhibit impaired sensorimotor gating [89]. Further research is needed to (1) assess the effective presence of a sensorimotor gating deficit in tic-related disorders, preferentially in studies made in human subjects, (2) elucidate the effective pathophysiological role of GABAergic interneurons and altered network connectivity in determining a deficit in sensorimotor gating processes and (3) evaluate the effectiveness of indirect markers of sensorimotor gating process, such as PPI, as indicators to test treatment effectiveness in tic-related disorders.
